# Letter from a Subscriber

**Published:** 1916-01

**Authors:** S. A. Visanska

**Affiliations:** Atlanta, Ga.


### LETTER FROM A SUBSCRIBER.

Editor Journal-Record of Medicine:

Dear Sir: Will you kindly give me space as an old subscriber to bring to your attention a few points in regard to the A. M. A. Journal published in Chicago? I received one of their regular letters, dated Dec. 2, 1915, also a copy of the Journal.

In the course of this letter they undertake to congratulate me on what they term, "The splendid spirit of co-operation I am showing by maintaining membership in your County and State Medical Organizations." The letter includes a spiel to become a fellow of the American Medical Association, and offers a special pecuniary inducement to this end. Then follows an exhaustive boost for the Journal, which is to be thrown in with a fellowship in the association for the grand sacrifice price of $5.00. The letter next refers to the amount of space devoted in the Journal to the methods of quacks and pretenders with their nostrums and fake cure. It finally closes with the claim that the Journal is, "Well nigh indispensable to the active man. Fill in and return your application blank today."

Let's dissect this letter and then take up a pamphlet enclosed with it. In the first place, whoever got up this letter appears to have missed his calling in not joining Barnum & Bailey Circus. It is a fine spiel, but is this the kind of letter to send out to physicians from what is supposed to be the highest Medical Organization in the country? A spiel, if you please, from one end to the other, and more than anything else it reminds us of the fellow in the circus who offers special inducements for remaining for the concert.

The pamphlet enclosed with the letter, unless I have misinterpreted it, means that the New York State Journal of Medicine and the A. M. A. Journal do not think it proper for a physician to subscribe to the New York Medical Record, Therapeutic Gazette, or other Journals carrying the adds of Angiers Emulsion, Camphor-Phenique, Sal Hepatica, Pasadyne, and others. Just in passing here, let me say a word in regard to the Pasadyne people, although they need no defense from me or any one else. Pasadyne is made in Atlanta, however, and I happen to know the house that makes it. If there are more honest, conscientious men anywhere I do not know them. Certainly I have not met them in my own profession. In addition to this I have used Pasadyne for a number of years, and I have gotten from it the results they claim for it.

In this connection I would like to show the inconsistency of the A. M. A. Besides the wonderful spiel they sent out to catch suckers, they carry an advertisement on page 6 of the edition of Aug. 28th, 1914, for Henry K. Wampole & Company in which the physician is advised if he "expects definite results he should prescribe" the Henry K. Wampole products.

The writer has nothing to say against Wampole or his preparations. I sold them when I was in the drug business, but I want to call to the attention of the A. M. A. the fact that this same company has had a Cod Liver Oil preparation on the market for years, that it has a red wrapper on the outside, that it retails for $1.00, but cut rates at about 73 cents in Atlanta. Now the A. M. A. would probably not endorse this preparation of Cod Liver Oil, which the Pure Food and Drug Act forced to adopt the name of Ext. of C. L. O., but they tell us in the advertising columns of the Journal that to get definite results, etc., we should prescribe these preparations. I have sold hundreds of bottles of C. L. O. and so far as I know it is all right. I am seeking only to show the absurd inconsistency of the organ of the A. M. A.

On page 15 of the same issue of the Journal an add reads: "A satisfactory summer food. In hot weather Bordens Eagle Brand Condensed Milk has a great advantage over ordinary dairy milk. It is not necessary to store in ice box for reasonable care in opening the can and keeping the contents covered and storing in a cool place will keep the food clean and wholesome. As a milk supply for summer use it has no equal, etc." Also see article by Dr. Wiley, Good Housekeeping, Nov. 15.

Now, as in the other case I am not saying that this is not what the manufacturer claimed for it, but my experience has taught me that there is only one satisfactory milk for infants, and that is Breast Milk. I have used in some cases the Eagle Brand and Baby Brand made by the same firm and have gotten good results. Look over ARCHIVES of PEDIATRICS and PEDIATRICS, two Journals devoted to diseases of children.

Their collaborators are among the best and most prominent men in the profession, yet these Journals carry advertisements which are attacked by the A. M. A. Journal.

If the Journal, from my viewpoint, was worth subscribing to, a letter like this would never have to be sent me. If the Journal from my viewpoint, was doing one-half the good claimed for it I would have been a subscriber long ago.

It is true the A. M. A. has exposed a number of frauds and quacks, but I blame each and every physician for using or tolerating such frauds as these. The A. M. A. does not take up the important point so necessary to the welfare of physician and public. The exposure of quacks and nostrums does not constitute everything, and who is the high muck to tell us what constitutes a quack or a nastrum? I had the honor of being graduated from the Philadelphia College of Pharmacy. I believe this College was able to give me a pretty good insight as to whether a preparation is a fake or not.

I am willing to give the A. M. A. credit for exposing some of these frauds. The Pure Food and Drug Act has also done good work, and should be, and I believe is, backed up by the majority.

If the A. M. A. would send out field secretaries to lecture on subjects looking to the betterment of our profession, if they would send men to the county societies, where only strife and ill-feeling exist. If they would devote a portion of the Journal, telling the members how to act towards each other, and to the public. If they would leave off the high and mighty scientific tone of their articles, and get down to plain facts, so that a majority of our profession could understand them. It occurs to me that if the A. M. A. and its organs would do even some of these things, they would find more followers among the rank and file of our profession.

Very respectfully,

S. A. Visanska, Ph.G., M. D.

Atlanta, Ga.

